# Correlation Between Age, the Development of Seizures, the National Institutes of Health Stroke Scale, the Modified Rankin Scale, and Discharge Status in Patients With Acute Hemorrhagic Stroke

**DOI:** 10.7759/cureus.95052

**Published:** 2025-10-21

**Authors:** Noha Mahmoud Youssef, Mohamed Shabana Hussein, Thel Su Hlaing, Khant Nyi Zaya, Myat Khant Wai, Mohamad Salah Shurbahji, Ahmed Elsayed Nour, Nada Breika, Marwa Harb, Rowa Murtada Ali Suliman, Sithu Bala, Esraa Samir Saleh Awadalla, Mohamed Bassem Elmoursi Abdou, Ahmad Adel Abdelhameed Muhammad, Sarah Haitham A Al-wardi, Mahmoud Ashraf Gaber Hani, Abdulmabod Omar

**Affiliations:** 1 Neurology Department, Qena University Hospitals, Qena, EGY; 2 Neurology Department, Mansoura International Hospital, Mansoura, EGY; 3 Internal Medicine Department, Nevill Hall Hospital, Abergavenny, GBR; 4 Velindre Cancer Centre, NHS Wales, Cardiff, GBR; 5 Medicine Department, University Hospitals Plymouth NHS Trust, Plymouth, GBR; 6 Faculty of Medicine, Ain Shams University, Cairo, EGY; 7 Fulbourn Hospital, Cambridgeshire and Peterborough NHS Foundation Trust (CPFT), Cambridge, GBR; 8 Peterborough City Hospital, North West Anglia NHS Foundation Trust (NWAFT), Peterborough, GBR; 9 General Practice, University Hospitals of Derby and Burton NHS Foundation Trust, Derby, GBR; 10 Older Person's Medicine, South Tees Hospitals NHS Foundation Trust, Middlesbrough, GBR; 11 Medicine, Lister Hospital, East and North Hertfordshire Teaching NHS Trust, London, GBR; 12 Emergency Medicine Department, University Hospitals Birmingham NHS Foundation Trust, Birmingham, GBR; 13 Emergency Medicine and Critical Care Department, Dr. Hassan Ghazzawi Hospital, Jeddah, SAU; 14 Psychiatry Department, Cygnet Hospital Wyke, Bradford, GBR; 15 Neurology Department, University Hospital Southampton, Southampton, GBR; 16 Faculty of Medicine, Al-Azhar University, Cairo, EGY

**Keywords:** hemorrhagic stroke, intracerebral hemorrhage, mrs, nihss, prognosis, seizures

## Abstract

Background

Seizures are a common consequence of acute hemorrhagic stroke, which has a high morbidity and fatality rate. It is yet unknown how they relate to age, the National Institutes of Health Stroke Scale (NIHSS), the modified Rankin Scale (mRS), and discharge status.

Methods

We conducted a cross-sectional study of 75 patients (aged 16-92 years) with acute hemorrhagic stroke admitted to the Neuropsychiatry Department of Qena University Hospitals, a tertiary care center serving a large catchment area in Upper Egypt. Data were collected over six months, from September 2024 to February 2025. Neurological impairment was assessed using the NIHSS, and functional outcome was measured using the mRS at admission and discharge. Statistical comparisons included paired t-tests and Wilcoxon signed-rank tests. Associations between seizures, age, stroke severity, and outcomes were also analyzed.

Results

Seizures were not correlated with age. During hospitalization, a noteworthy mean improvement in NIHSS (+0.97; t = 10.70 and p < 0.0001; W = 21.50 and p < 0.0001) was noted, suggesting neurological recovery. On the other hand, mRS remained at a median 3 (t = -0.51 and p = 0.6113; W = 275 and p = 0.9173), indicating no discernible change. There was no discernible change in NIHSS or mRS among patients who were released on demand (n = 10). Ten out of the 12 in-hospital fatalities had seizures, and the majority had moderate-to-severe strokes (NIHSS ≥9; mRS ≥3). Patients with very severe strokes (NIHSS 16-17; mRS = 5) and no recovery experienced two non-seizure fatalities. Admission NIHSS (1-17) and mRS (1-5) showed a broad variety of stroke presentations and recovery trajectories.

Conclusion

The baseline of stroke severity was the best indicator of a bad prognosis in acute hemorrhagic stroke, and seizure incidence was not age-dependent. Significant neurological recovery was recorded by NIHSS, but short-term functional improvement was not reflected by mRS. These results indicate that seizures are more common in fatal cases but secondary to the total load of strokes, underscoring the prognostic significance of baseline severity.

## Introduction

Acute hemorrhagic stroke remains a significant cause of morbidity and mortality worldwide, with clinical severity and functional outcomes varying widely across patients [1]. Hemorrhagic stroke includes intracerebral hemorrhage (bleeding within the brain tissue) [2] and subarachnoid hemorrhage (bleeding into the space between the brain and the surrounding membranes) [3], which is linked to high rates of disability and death. Despite advances in stroke care, outcomes of acute hemorrhagic stroke remain worse than those of ischemic stroke due to limited treatments and unpredictable course [4]. Seizures are a known side effect of acute hemorrhagic stroke that affects 6-15% of patients, especially those with significant hemorrhage volumes, cortical involvement, or lobar hematomas [5,6]. These seizures may be a contributing factor to secondary brain damage by decreasing neurological function, elevating intracranial pressure, and increasing metabolic demand [5]. Optimizing monitoring and treatment options requires the early identification of individuals who are at risk for seizures. However, there is still inconsistency among research regarding the clinical predictors of post-acute hemorrhagic stroke seizures. Few studies have examined how seizures interact with both neurological and functional outcomes (National Institutes of Health Stroke Scale (NIHSS) and modified Rankin Scale (mRS)) during hospitalization in low-resource settings. Some studies indicate no significant correlation between age and the severity of a stroke, whereas others identify younger age, cortical placement, and higher hematoma volume as risk factors [7,8]. Furthermore, there is ongoing controversy regarding the effect of seizures on mortality and functional results. While some studies contend that seizures impair prognosis, others primarily ascribe poor outcomes to the severity of the initial stroke [9,10].

A standardized method for measuring neurological disability in stroke patients is the NIHSS. It offers an objective assessment of the severity of the stroke upon admission in cases of acute hemorrhagic stroke and is closely linked to early outcomes, such as death and the possibility of complications like seizures. It has been demonstrated that higher NIHSS scores predict a bad prognosis and suggest more severe deficiencies, making it an essential tool for research and therapeutic decision-making [11].

Moreover, the most used tool for evaluating functional impairment following a stroke is the mRS. The mRS measures the patient's degree of independence in day-to-day activities, which provides supplementary data about long-term recovery and quality of life [12], whereas the NIHSS records neurological damage. mRS is commonly employed as a primary endpoint in clinical research for hemorrhagic stroke because it provides a straightforward, clinically useful summary of the global functional outcome. A thorough understanding of the neurological and functional consequences of acute hemorrhagic stroke may be obtained by examining it with the discharge status. In addition to guiding resource allocation and improving prognostic models, this integrated approach can shed light on the effects of seizures and other problems. Therefore, this study aims to determine whether seizure occurrence is associated with stroke severity and outcomes independent of age.

## Materials and methods

Study design and setting

This was a cross-sectional study conducted at the Neuropsychiatry Department of Qena University Hospitals, a tertiary care center serving a large catchment area in Upper Egypt. Data were collected over six months, from September 2024 to February 2025.

Study population

A total of 75 patients diagnosed with acute hemorrhagic stroke were included in the study. All patients were admitted to the inpatient neurology ward or evaluated in the neurology outpatient clinic at Qena University Hospitals during the study period.

The inclusion and exclusion criteria are presented in Table 1.

**Table 1:** Inclusion and exclusion criteria

Inclusion criteria	Exclusion criteria
Male and female patients aged 16 years and older	Patients aged less than 16 years
Confirmed diagnosis of hemorrhagic stroke based on clinical presentation and radiological evidence	History of epilepsy before the stroke
	Diagnosis of transient ischemic attack or cerebral venous thrombosis
	Patients with drug-related seizures
	Evidence of structural brain lesions unrelated to stroke (e.g., brain tumors, infections, or functional neurological disorders)

In light of the inclusion criteria, the following variables were considered for the study: age, recorded as a continuous variable and later stratified into subgroups for analysis, development of seizures, categorized as a binary variable (yes/no) during hospitalization, and NIHSS and mRS scores, recorded at admission and at discharge to assess changes in stroke severity and functional outcome.

Data collection

The sample size was based on the consecutive sampling of all eligible patients presenting during the data collection period. All patients who met the inclusion and exclusion criteria were registered. After obtaining informed consent, all patients underwent a comprehensive medical history, physical examination, and neurological assessment. Stroke severity was assessed using the World Health Organization (WHO) criteria for stroke diagnosis and imaging-based confirmation criteria. The NIHSS was taken on admission and at discharge of participants. Functional outcome and disability assessment were also taken using the mRS on admission and at discharge, while seizure assessment in participants was based on clinical observation and criteria established by the International League Against Epilepsy (ILAE).

Ethical considerations

The study protocol was reviewed and approved by the Institutional Review Board (IRB)/Ethics Committee of Qena University Hospitals (approval number: 902-NAP020; date: 2/8/2024). Before enrolment, all subjects provided written informed consent. The Declaration of Helsinki's ethical guidelines were followed when conducting the study [13]. All participant data was anonymized to ensure confidentiality, and biological samples were kept in a secure location with limited access.

Statistical analysis

Data were analyzed using IBM SPSS Statistics for Windows, V. 26.0 (IBM Corp., Armonk, NY, USA). Descriptive statistics were used to summarize patient characteristics. Continuous variables such as age, NIHSS score, and mRS score were expressed as mean ± standard deviation (SD) and median with interquartile range (IQR). For paired comparisons of NIHSS and mRS scores at admission and discharge, both the paired t-test (for parametric data) and the Wilcoxon signed-rank test (for non-parametric confirmation) were applied. A p-value of <0.05 will be considered significant. Discharge status was categorized as improved, on-demand discharge, or death. Because of the limited sample size and imbalance between groups, descriptive statistics (mean ± SD, cross-tabulations with seizure occurrence, and NIHSS severity categories) were reported for these outcomes.

## Results

Patient characteristics

This cross-sectional study consisted of 75 patients who had been diagnosed with acute hemorrhagic stroke (Table 2). The study population ranged in age from 16 to 92 years old, with a mean age of 62.4 ± 13.7 years. Most of the patients were in the 60-80-year age range. Of the 75 patients, 52 patients (62.73 ± 14.15 years) did not experience seizures while in the hospital, while 23 patients (64.56 ± 17.3 years) did. This difference was not statistically significant (t(36) = 0.45; p = 0.657), indicating that age was not associated with the development of seizures in this cohort. This implies that in individuals with acute hemorrhagic stroke, age may not be a reliable independent predictor of the onset of seizures.

**Table 2:** Comparison of age between patients with and without seizures Statistical test: independent samples t-test; significance threshold: t(df) < 0.05

Variable	Seizure group (n = 23)	No seizure group (n = 52)	t(df) test statistics
Mean age (years)	64.56 ± 17.3	62.73 ± 14.15	t(36) = 0.45; p = 0.657
Proportion of total (%)	30.66%	69.33%	

NIHSS

The NIHSS was used to evaluate 75 individuals both upon admission and upon release. At admission, the median NIHSS score was 8.0 (IQR 6.0-11.5), while the mean score was 8.81 (SD = 4.37). The median NIHSS at discharge was 7.0 (IQR 5.0-11.0), while the mean NIHSS dropped to 7.84 (SD = 4.40). This indicates that neurological status has generally improved. The significant score decline was validated by paired statistical analysis. A mean difference of almost 0.97 between admission and discharge scores was found using the paired t-test (t = 10.70; p < 0.0001). This finding was further validated by the non-parametric option, the Wilcoxon signed-rank test (W = 21.50; p < 0.0001). According to these results, patients' overall quality of life improved statistically significantly while they were in the hospital.

Although complete recovery to no symptoms was uncommon, only two patients received a score of 0; the majority of patients had moderate strokes (NIHSS 5-15) upon presentation and demonstrated noticeable improvement by discharge. Many patients showed positive recovery among those who initially scored higher, improving to the small stroke category (1-4). Only one patient had a severe stroke score (≥21) without recovery, and a small minority remained in the moderate-to-severe range (16-20) at both admission and discharge, with little to no improvement. These results demonstrate that whereas individuals with moderate strokes frequently recover somewhat, those with more severe strokes have a harder time making a noticeable neurological improvement (Table 3).

**Table 3:** NIHSS scores at admission and discharge Paired t-test (t = 10.70; p < 0.0001); Wilcoxon signed-rank test (W = 21.50; p < 0.0001) Statistical test: independent samples t-test; significance threshold: t < 0.05 NIHSS: National Institutes of Health Stroke Scale; IQR: interquartile range

Measure	Mean (SD)	Median (IQR)
NIHSS at admission	8.81 (4.37)	8.0 (6.0-11.5)
NIHSS at discharge	7.84 (4.40)	7.0 (5.0-11.0)

mRS

Functional impairment was evaluated at admission and discharge using the mRS. At release, the mean mRS score was 2.91 ± 1.64, whereas at admission, it was 2.84 ± 1.17. With a minor reduction in the IQR from 2-4 at admission to 2-3.5 at discharge, the median mRS stayed constant at 3. The mRS scores did not alter statistically significantly between admission and discharge, according to a paired t-test (t = -0.51; p = 0.61). The non-parametric Wilcoxon signed-rank test (W = 275.00; p = 0.9173) also revealed no discernible change in mRS scores. These findings imply that, despite slight individual variations in impairment scores, the sample as a whole did not see any consistent or statistically significant functional improvement during the hospital stay (Table 4).

**Table 4:** Comparison of mRS scores on admission and discharge Paired t-test (t = -0.51; p = 0.61); Wilcoxon signed-rank test (W = 275.00; p = 0.9173) Statistical test: independent samples t-test; significance threshold: t < 0.05 mRS: modified Rankin Scale; IQR: interquartile range

Measure	Mean (SD)	Median (IQR)
mRS at admission	2.84 (1.17)	3 (2-4)
mRS at discharge	2.91 (1.64)	3 (2-3.5)

Descriptive analysis of seizure vs. non-seizure acute hemorrhagic stroke patients

Out of 75 patients, a subgroup of 10 patients with acute hemorrhagic stroke were discharged on demand, with no significant improvement in NIHSS (before 10.40 ± 3.75 and after 10.20 ± 3.77) or mRS (before 2.90 ± 0.88 and after 2.8 ± 1.05) scores observed during hospitalization. The majority (90%) had unchanged neurological and functional status at discharge compared to admission, indicating limited short-term recovery. Among the three patients (30%) who experienced seizures during their hospital stay, NIHSS and mRS scores also remained stable, suggesting that seizure occurrence did not influence discharge decisions or short-term outcomes in this study.

In a subgroup of 12 patients who died, 10 experienced seizures during hospitalization, while two did not. The seizure group included patients across a broad NIHSS range (1-22), but most had moderate-to-severe strokes (NIHSS ≥9) and high functional disability (mRS ≥3). The mean NIHSS before administration was 11.08 ± 6.29 and mRS 3.92 ± 1.31. The two patients without seizures both had very high NIHSS scores (16-17) and severe disability (mRS = 5) at admission. Despite the absence of seizures, they still died, with no improvement in neurological status before discharge. Ultimately, all 12 patients died (mRS = 6) at discharge. This supports evidence that seizures often complicate larger or cortical strokes and may worsen outcomes. But in the case of two patients, result shows that a very severe stroke may cause mortality, even without seizures.

In a subgroup of 56 patients (10 have seizures) who show improvement, ranging in age from 16 to 92 years, stroke severity measured by NIHSS scores on admission varies widely from mild (score of 1) to severe (score of 17). Similarly, disability measured by the mRS on admission ranges from minimal to severe levels (scores 1-5). Across the cohort, there is a consistent pattern of clinical improvement from admission to discharge, as reflected by decreases in both NIHSS (8.00 ± 3.74 to 6.66 ± 3.44) and mRS (2.58 ± 1.06 to 2.23 ± 1.01) scores. Discharge NIHSS scores generally fall between 0 and 14 and mRS scores between 0 and 4, indicating reduced stroke severity and disability by the time of discharge. The data show considerable heterogeneity in stroke severity and disability, highlighting the variability in patient presentations and recovery trajectories. Despite this, all patients were discharged with an improved status, underscoring effective clinical management across different ages and stroke severities.

## Discussion

Age did not correlate with seizure occurrence in this cross-sectional research of 75 patients with acute hemorrhagic stroke (16-92 years old), indicating that seizure risk in acute hemorrhagic stroke is spread across the adult age spectrum within our cohort. Other work also correlates with this conclusion that age effects are inconsistent. Many analyses find no consistent effect of age on early seizures; the results vary depending on whether the study looks at early vs. late seizures, hemorrhage location (cortical/lobar), volume, and surgical intervention. However, some intracerebral hemorrhage studies and meta-analyses list younger age as a risk factor for late seizures and include age <65 as part of predictive scores (e.g., the CAVE score for late seizures) [14,15].

A noteworthy recovery in neurologic impairments during hospitalization was confirmed by the significant mean improvement of 0.97 points in NIHSS from admission to discharge (paired t-test: t = 10.70 and p < 0.0001; Wilcoxon signed-rank test: W = 21.50 and p < 0.0001). In contrast, there was no significant change in the median mRS, which stayed at 3 (t = -0.51 and p = 0.6113; W = 275 and p = 0.9173), indicating that functional impairment did not significantly improve. The NIHSS assesses the effects of a stroke on consciousness, language, neglect, visual field loss, extraocular movement, motor strength, ataxia, dysarthria, and sensory loss, and ratings for each item are scored on a 3- to 5-point scale, with 0 being normal and higher scores indicating greater severity. The mRS is a one-item measure that assesses the degree of disability or dependence in daily activities in stroke patients, and scores range from 0 to 5, with higher scores indicating greater disability.

The mRS is a global functional disability scale that frequently changes more slowly or requires greater improvements to demonstrate a shift [16] than the NIHSS [17], which examines neurologic deficits and is more sensitive to short-term changes in impairment. Numerous studies point out that early improvements in NIHSS (neurological exam scores) do not always result in better mRS after discharge, particularly if follow-up is brief and the mRS baseline is moderate (mRS 3) [17]. We found no significant improvements in NIHSS or mRS in the subset discharged on demand (n = 10), supporting the idea that patients who leave too soon have stopped recovery, which is in line with observations that discharge against medical advice (DAMA) is linked to lower short-term outcomes and incomplete recovery [18].

Ten of the 12 patients (who died) experienced seizures while in the hospital, with NIHSS ranging from 1 to 22, and the majority of them had moderate-to-severe strokes (NIHSS ≥9; mRS ≥3). Regardless of seizure occurrence, two non-seizure deaths had very severe stroke (NIHSS 16-17; mRS = 5) with no neurological recovery, highlighting the fact that a high initial stroke severity predicts a bad prognosis. Several studies have linked early seizures following intracerebral hemorrhage to a poorer in-hospital course (deterioration) and a greater fatality rate [19]; however, the association is complicated by initial severity (high volume, low Glasgow Coma Scale score, high NIHSS) and cortical placement. Seizures frequently occur in patients with larger/lobar cortical hemorrhages, according to several analyses, and early seizures can be indicators of more severe injury and predict a worse outcome. However, some studies find that seizures are not a reliable indicator of long-term outcome in all cohorts when baseline severity and imaging markers are taken into account [6,20]. Our results complement and vary from various facets of the body of current literature because hemorrhagic stroke presentations and recovery trajectories are heterogeneous, as evidenced by the wide range of admission NIHSS (1-17) and mRS (1-5) in the improving sample (n = 56, including 10 with seizures) so seizures considered as an independent prognostic factor which need further studies to understand the connection between seizures and outcomes in acute hemorrhagic stroke and seizure occurrence should be considered a severity marker which needs immediate interference.

Limitations

It is important to recognize the numerous limitations of this study. First, the results' applicability to larger or more varied populations is limited by the single-center design and the very small sample size (n = 75). Second, the study only identifies correlations seen during hospitalization; due to its cross-sectional methodology, it is unable to prove a causal link between seizures, stroke severity, and outcomes. Third, because follow-up was restricted to the hospital stay, it was not possible to evaluate the prevalence of delayed death, long-term functional recovery, or late seizures. Fourth, as not all patients had access to continuous electroencephalography (EEG) monitoring, the reliance on clinical observation for seizure identification may have resulted in the underreporting of subclinical or electrographic seizures. Fifth, a subset of patients may have experienced bias due to DAMA, which limited findings about their long-term results and truncated their recovery data. Finally, the interpretation of seizure risk and prognosis may be complicated by the lack of systematic analysis of crucial factors such as hematoma volume, location, intraventricular extension, and antiepileptic medication usage.

Recommendation

To further understand the connection between seizures and outcomes in acute hemorrhagic stroke, future research should overcome the presented limitations. Larger sample numbers from multicenter prospective trials would enhance external validity and enable subgroup analysis by treatment strategy, age, and stroke subtype. More accurate incidence estimates and improved seizure identification, especially for non-convulsive or subclinical seizures, might result from incorporating continuous or extended EEG monitoring. To assess late seizures, long-term functional recovery, and survival outcomes, follow-up must be extended beyond hospitalization. Since hematoma volume, location, and intraventricular extension are significant predictors of seizures and prognosis, future studies should also incorporate these specific neuroimaging characteristics. Furthermore, evaluating the function of therapeutic or preventative antiepileptic medication use may aid in figuring out how these drugs affect patient outcomes. Lastly, health systems should create DAMA-minimization plans that include social support and patient education to guarantee proper inpatient rehabilitation and lower the chance of adverse outcomes.

## Conclusions

Age did not correlate with seizure risk in acute hemorrhagic stroke. There was a discrepancy between neurologic recovery and functional result after hospitalization, as evidenced by the improvement in NIHSS scores but not in mRS. Patients with severe strokes accounted for the majority of fatalities, suggesting that baseline severity is the primary predictor of a poor outcome.
